# Artificial intelligence-based organizational human resource management and operation system

**DOI:** 10.3389/fpsyg.2022.962291

**Published:** 2022-07-22

**Authors:** Yang Yang

**Affiliations:** School of Management and Economics, North China University of Water Resources and Electric Power, Zhengzhou, China

**Keywords:** human resources, artificial intelligence, operational systems, management operations, data mining

## Abstract

The trend of globalization, marketization, and informatization continues to strengthen, in today’s development environment, how to seize the opportunity and obtain a competitive advantage in human resources is an important issue that needs to be explored. Human resource management refers to the effective use of relevant human resources inside and outside the organization through management forms under the guidance of economics and humanistic thinking. It is a general term for a series of activities that ensure the achievement of organizational goals and the maximization of member development. With the rapid development of society and economy, the competition between enterprises has intensified. If an enterprise wants to adapt to social development, it is necessary to strengthen the internal management of the organization. The internal management also needs to rely on human resource management. The purpose of this paper is to study an organization’s human resource management and operation system based on artificial intelligence. It expects to use artificial intelligence technology to design the human resource management system and to improve the quality of employees to make the enterprise develop toward a more scientific and reasonable method. It uses artificial intelligence technology to mine the relevant data of enterprises, understand the situation of enterprises in a timely manner, and adjust unreasonable rules. This paper establishes a dynamic capability evaluation model and an early warning model for human resource management and further studies the improvement approach based on human resource management. This paper analyzes the application, feasibility, and practical significance of data mining technology in human resource management systems. It focuses on the commonly used algorithms in the field of data mining and proposes specific algorithm application scenarios and implementation ideas combined with the needs of human resource management practices. The experimental results of this paper show that the average working life of incumbent employees is 3.5 years, the average length of employees who leave the company is 5 years, and some employees are 5–6 years old. From this data, it can be seen that the average number of years of on-the-job employees is short, and the work experience has yet to be accumulated.

## Introduction

The rapid development of knowledge economy has broken the barriers of information transmission and brought new business ideas and methods at the same time, and the change of thinking mode has made people more and more aware of the importance of human resources. It has been proved by practice that the production of human resources plays a decisive role in the development of production and guarantees the implementation of the organization’s business strategy. With the continuous progress of society, human resource management faces many problems, including the challenges of structural diversification and competition diversification. In the era of knowledge economy, the advanced western management thoughts have a strong impact on the traditional management knowledge system, and the importance of organizational human resources has made its status in organizational management increasingly promoted. These are the reasons for adopting new methods. Studying the dynamic capabilities of human resources can provide an effective method for human resource management to judge the effectiveness of organizational management behavior and job suitability standards, and it provides intellectual support for enterprises. The use of AI is a way to help companies dig deeper into the treasures of HRM information and improve their efficiency and core competitiveness. Modern management puts forward higher requirements for enterprise human resource management mode, so it is very suitable to implement computerization of enterprise human resource information processing.

The tremendous advances in information and communication technology (ICT) and the emergence of the knowledge economy pose great opportunities for companies to meet their business objectives. Majumder focused on an in-depth understanding of current human resource management practices and their impact on employee satisfaction. For the study, he selected 100 bank employees, of which 88 employees responded correctly, with a response rate of 88%, and the questionnaire was compiled using a point scale. Point scale (point scale), also known as “point scale,” is a test scale with points as the scoring unit. Its advantages are that the scoring is accurate and simple. Research shows that employees are not equally satisfied across all human resource management dimensions. Most employees were dissatisfied with compensation packages, followed by rewards and incentives, career development, training and development, management style, job design, and responsibilities. Therefore, the quality of these human resource management dimensions should be improved (Majumder, 2017). Guest proposed an approach to human resource management that prioritizes well-being and positivity. He provided evidence to support practice choices and argued that these practices also have the potential to improve individual and organizational performance (Guest, 2017). Crowley investigated to see whether HRM practices implemented as complementary ‘bundles’ or ‘systems’ in Irish manufacturing and service sector companies are more effective than those implemented separately. HRM bundles associated with merit control and appraisal, learning, exchange, and decision-making, involvement, and delegation were all found to be actively related to creativity in both firms in the manufacturing and service sectors. In conclusion, HRM practices are important for company innovation when applied together rather than in isolation (Crowley and Bourke, 2017). Sandra drew on a resource-based perspective and used suppositions about a company’s CSP strengths from an objective third-party database. The study results show that human resource management and creativity are key capabilities because they enable the creation and enhancement of other capabilities (Rothenberg et al., 2017). Organizational learning is becoming increasingly important for strategic updating. Agile organizations are especially successful in the current environment, where companies need to be efficient and adapt to change. Organizational Learning Organizational learning refers to the various actions that organizations take around information and knowledge skills to achieve development goals and improve core competitiveness. Diaz-Fernandez took a constructive view. He emphasized the distinction in terms of what is learned between sales and delivery units in the case of different HRM measures of risk and human energy profiles. The experimental results point to a mediating role of human capital between HRM practices and learning (Diaz-Fernandez et al., 2017). Although these theories discuss human resources, they do not use artificial intelligence technology.

AI is a hot technology nowadays, and there are many studies on it. Burton wanted to develop a curriculum to introduce AI. He offered specific suggestions for integrating AI into the curriculum and how to teach a stand-alone course on the ethics of AI (Burton et al., 2017). Bin provides a conceptual framework for integrating statistical ideas with human input into AI products and research. These ideas include the principles of experimental design for randomization and local control. He discussed the connection of these principles in self-driving cars and automated medical diagnostics (Bin and Kumbier, 2018). Polina outlined the next generation of artificial intelligence and blockchain technologies. It enables patients to control and monetize their personal data through new tools, as well as incentives for continuous health monitoring (Polina et al., 2018). Organizational Learning Organizational learning refers to the various actions that organizations take around information and knowledge skills to achieve development goals and improve core competitiveness. Although these theories have expounded artificial intelligence, their integration with human resource management is less practical.

With the progress of the times, people pay increasingly attention to resources. In the current fierce competition environment, companies must strengthen the management of employees within the organization if they want to stay at the forefront of the industry. Experiments show that the incumbency rate of management positions is as high as 80%, the turnover rate is only 20%, and the incumbency rate of market positions is 71%. It shows that the turnover of different positions is different; the incumbency rate of low salary is 67%, the incumbency rate of general salary is 79%, and the incumbency rate of high salary is 88%. It shows that salary is an important factor affecting employees, and retaining employees can start from salary. The turnover rate without promotion within 3 years is 25%. The turnover rate of job promotion is 10%, indicating that long-term lack of promotion is an important factor leading to employee turnover.

## Human resource management operation system method

### Human resource management

With the development of the times, the connotation of human resources continues to expand. However, “human resources” was first proposed in the middle of the last century. At that time, scholars believed that human resources were a special kind of resources, which must be stimulated by relevant incentive mechanisms, thus bringing their due value (Grzonka et al., 2017; Lee et al., 2017a). Enterprises believe that human resource management refers to the deployment requirements and macro directions according to the enterprise development strategy. It fully mobilizes the enthusiasm of employees to work, stimulates the potential of employees, and finally achieves the strategic goals of enterprise development. People are the active force of social development. If people cannot develop their knowledge and skills, the development of all new things will be hindered. Human resource management has the ability to predict the human resources of the organization screen personnel according to the predicted conditions, integrate compensation, and optimize the construction of the entire organization (Lee et al., 2017b; Youssef et al., 2017). Organization optimization is the planner of the overall layout of the enterprise; organization optimization is the founder of enterprise system; organization optimization is the promoter of enterprise reform; organization optimization is the cultivator of enterprise cultural heritage.

There is a lot of content in human resource management, and here we briefly describe performance compensation and training (Labovitz et al., 2017). Performance refers to the stage results achieved by employees according to their duties and the behavioral performance in the process of achieving the stage results. What we call performance management is a series of measures taken to achieve performance (Agrawal et al., 2017; Price and Flach, 2017). Performance management forms an objective and fair closed-loop management mechanism to motivate employees to make work behaviors that are beneficial to the development of the enterprise, to achieve organizational goals and achieve closer integration of organizational and employee performance (Ghoson and Faris, 2021). The performance management system mainly includes performance planning, including performance implementation, performance appraisal, performance communication and feedback, and performance improvement.

**Figure 1:**
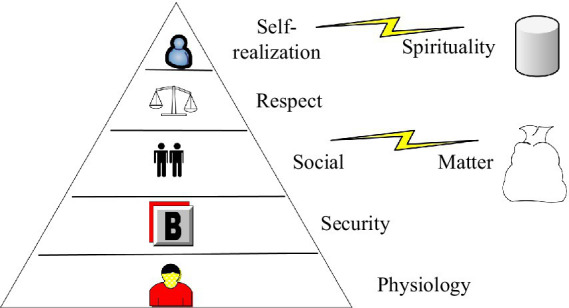
Distribution of Maslow’s hierarchical model of needs.

The property owner is separated from the laborer, and the remuneration paid by the owner to the laborer is called salary, and we now call it the value created (Shivam et al., 2020). With the progress of the times, people are increasingly concerned about things other than external compensation, that is, inner feelings, which we call intrinsic compensation. This is an important reason for the internal imbalance of employees (Belizon et al., 2017). External remuneration refers to salary bonuses, allowances and subsidies, insurance benefits, etc., including monetary and nonmonetary returns. We can think of this kind of compensation as human needs. According to Maslow’s Hierarchy of Needs, we can divide it into different categories, and its distribution pattern is shown in Figure 1.

**Figure 2:**
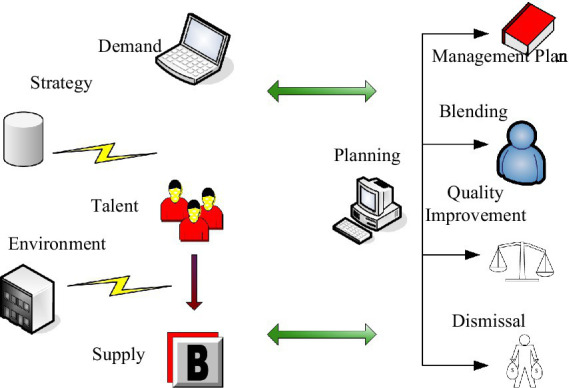
Enterprise talent development planning model.

The continuous development of enterprises is inseparable from the progress of employees. Compared with recruiting new employees, training existing employees in line with the company’s development strategy is one of the lowest-cost and fastest effective human resource management measures. According to the actual situation, it is also verified that training and development is an important means to improve the comprehensive quality of employees. Corporate training enables employees to quickly understand the corporate culture and have a general understanding of the company’s work. In this process, the company can also conduct a comprehensive evaluation of employees and match employees to jobs. Conventional enterprise training management includes a four-step process of training needs analysis, training plan formulation, organization and implementation, training effect evaluation, and feedback. Development is not the same as training. Development needs to improve the creativity and skills of employees, coordinate the personal goals of employees and corporate goals, and enable employees to develop healthily and quickly in the enterprise. Due to the hysteresis of the training effect and the differences and personalization of individual employees in their work, scientific and objective evaluation of the training effect is the most difficult part of the training management process (Cooke, 2017; Ones et al., 2017). Figure 2 shows the enterprise talent development planning model.

### Brief introduction of artificial intelligence

**Figure 3:**
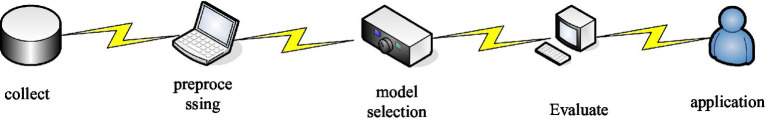
Relevant flow chart of data mining.

Data mining is a continuous process. When using this method, it is first necessary to determine the mining objects and targets. In the process of data mining, we need to classify the objects according to the rules. The theme of this paper is human resource management, so we can subdivide the goals into employee-related elements such as performance and turnover in the data mining process, and then select the appropriate target set according to the relevant target groups. Figure 3 shows the relevant flow chart of data mining.

The multiple regression algorithm belongs to the primary artificial intelligence algorithm, and its principle is very simple, so it is very widely used in practical applications. In regression analysis, if there are two or more independent variables, it is called multiple regression. In fact, a phenomenon is often associated with multiple factors, so it is more effective to predict or estimate with multiple independent variables than with only one independent variable. We briefly introduce it.


(1) $$g_{\varepsilon }\left(a\right)=\varepsilon_{0}a_{0}+\varepsilon_{1}a_{1}+\varepsilon_{2}a_{2}+\cdots +\varepsilon_{k}a_{k}$$

In formula (1), $g_{\varepsilon }\left(a\right)$ represents the multiple regression value, k represents its dimension, and $\varepsilon_{k}a_{k}$ represents the sample set.


(2) $$g_{\varepsilon }\left(A\right)=\varepsilon *A$$

In formula (2), A represents a dimension matrix.


(3) $$Y\left(\varepsilon_{0},\varepsilon_{1},\varepsilon_{2},\cdots ,\varepsilon_{k}\right)=\sum \left(g_{\varepsilon }\left(a_{0},a_{1},a_{2},\cdots ,a_{k}\right)-b_{p}\right)$$

In formula (3), we take the mean difference as the loss function and then fit it by minimizing it. The loss function is an operation function used to measure the difference between the predicted value f(x) of the model and the real value Y. It is a nonnegative real-valued function, usually represented by L [Y, f(x)]. The loss function, the smaller it is, the more robust the model is.

Combined with human resource management, we can use the linear regression method to establish a human resource demand forecast model. The regression algorithm has many data features, and it is an algorithm used to evaluate the relationship between the dependent variable and multiple independent variables. In a human resource management system, the emergence of a decision will affect multiple factors. However, the algorithm needs to determine whether there is a certain linear correlation between the influencing factors of human resources and the prediction target in the actual application process.

From an enterprise’s point of view, human resource management is mainly determined through experience and needs, but its basis is a relatively subjective element, and there are no specific rules to follow. Through the linear regression algorithm, the relevant factors that affect the human resource demand of the enterprise can be accurately found, to help the enterprise manager to more clearly define the amount of human resources required by the enterprise, make a more scientific and reasonable allocation method, and lay a good human resource for the development of the enterprise. Base.

K-means algorithm belongs to the clustering algorithm and is widely used in the field of data mining. We can also use it in the human resource management module to establish a performance appraisal model. It helps enterprises to construct the correct decision-making method, realize the objectivity of assessment, and realize scientific management.


(4) $$\mathrm{Support}=\left(\mathrm{a,b}\right)=Q\left(\mathrm{a,b}\right)=\frac{\vartheta \left(a\cup b\right)}{k}$$

(5) $$\mathrm{Confidence}=\left(\mathrm{a,b}\right)=Q\left(\mathrm{a,b}|a\right)=\frac{\vartheta \left(a\cup b\right)}{\vartheta \left(a\right)}$$

In the above function expression, a,b represents two different events, formula (4) represents support, and formula (5) represents confidence.

Usually, we apply the K-means algorithm to the employee performance appraisal management module of the human resource management system. Realization needs to select indicators related to employee performance appraisal according to the actual situation of the enterprise. Such indicators as innovation and team capability are then assigned points based on their importance. The total score of the indicator represents the overall performance level of the employee. The construction of this employee performance appraisal model can help enterprises to abandon the traditional subjective decision-making method and realize the objectivity of employee appraisal and evaluation. It is an important auxiliary tool for modern enterprises to achieve scientific management.

The Naive Bayes algorithm is a classification method based on the Yebes theorem and the assumption of independence of feature conditions, and it also belongs to a class of data mining algorithms.


(6) F(R)(a,b)>F(t)(a,b)

In the above function expressions, F(R) and F(t) are not the probability functions we often say, they refer to Bayesian probability, which is what we often say the posterior probability.

**Figure 4:**
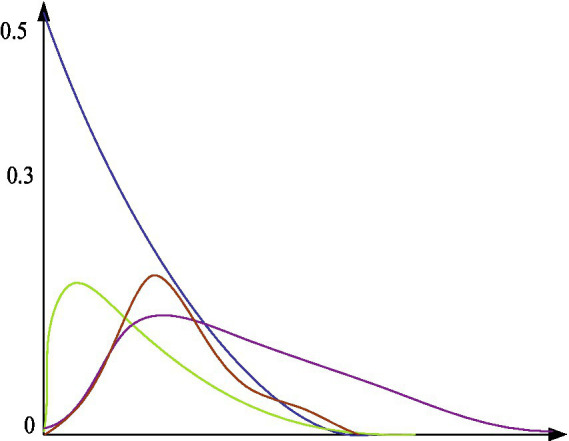
Common models of conditional probability.

Figure 4 shows a common model of conditional probability.

**Figure 5:**
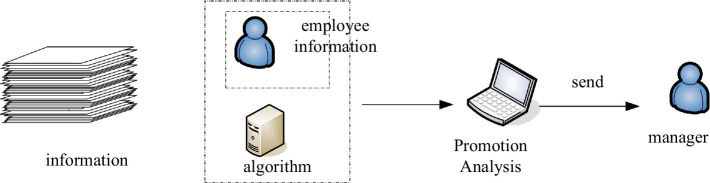
Employee promotion phase algorithm-related model.

In practical applications, for a given training set, we need to independently assume the joint distribution probability according to the feature conditions, and then calculate the posterior probability according to the model. When managing the human resource structure based on the employee personal information in the human resource management system, we first need to select representative employee characteristics, set the minimum support and confidence for mining, and discover the implicit strong correlation in the data. For example, dig out the unreasonable aspects of the degree structure and professional title structure of the enterprise, determine some problems in the current workforce structure, and realize the optimization of the workforce. In addition, we can make promotion decisions based on this decision-making model. Through the algorithms, we can mine the matching degree between the current position of the company’s employees and other positions, so as to provide scientific matching information when the company’s personnel changes, and its model structure is shown in Figure 5.

## Human resource management operation system experiment

### System structure design

**Figure 6:**
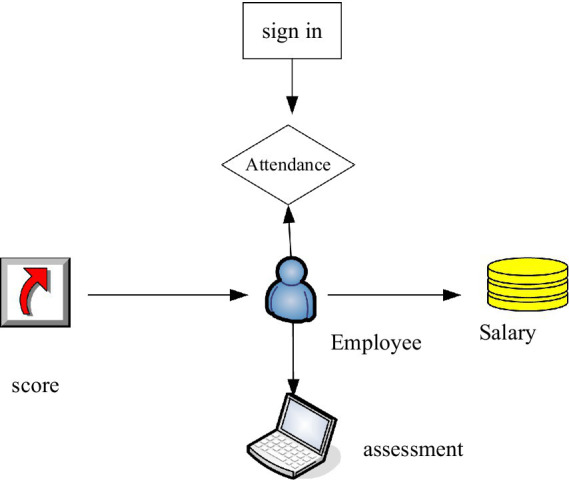
E-R Diagram of human resource management system.

Database design is an important part of enterprise human resource probabilistic system design. In the database design, it is necessary to carefully study the specification documents and design documents, clarify the content of the database design, and then select the appropriate database management system according to the content. E-R is a diagram used to model data and identify the relationship between functions and data. Figure 6 is the E-R diagram of the human resource management system.

Employee information needs to be recorded in the system. According to the system settings in Table 1, the employee number is generally set to a char type with a length of 10 digits. This number will be given when the employee joins the job. This number is unique and cannot be changed later. The name is set to a variable-length char type, and the length can be up to 20 characters. Gender settings can input 2-bit length char types, and some information settings can input 12-bit length char types. The first and second digits are the number of the department, the third digit is the incremental post code of the department, and the fourth digit is the number of employees in this position. In fact, the employee number is the most important among these information, and the employee information can be searched according to the number in the system. Therefore, in the data registration form, except for the employee number, which is not allowed to be empty, all other information can be empty.

**Table 1:** Basic information of employees.

Category	Employee number	Employee name	Gender	Department	Phone number	Remarks
Type	Int	Char	Char	Char	Char	varChar
Size	8	10	2	12	12	50
Properties	No	Yes	Yes	yes	yes	yes

### Database logic design

This article creates each table in the database according to the designed entity E-R diagram. It establishes different data tables according to the relevant information required by the enterprise to investigate and analyze the relevant situation of employees. The more important data table structure is given below.

According to the data in Table 2, we briefly introduce some relevant information and structures. According to the E-R diagram, the department number is generally set to a varChat type with a length of 5 digits. Because the enterprise has different departments, a fixed number is given when the department information enters the system, and the number is fixed and no one has the right to change it. The name of the department is generally set to a varChat type with a length of 10 digits. The name and number are different. The number cannot be changed, but the name can be changed. As long as it is within a reasonable character, it can be changed. The manager-related information of the department is generally set to a varChat type with a length of 20 digits, and the manager’s area of responsibility can be briefly described in the system. The address of the department is generally set to a varChat type with a length of 20 digits, and the telephone of the department is generally set to a varChat type with a length of 50 digits. According to the above department information table, the number of the department is the most important, and the relevant information of the department can be obtained in the system according to the department number.

**Table 2:** Departmental table related structure.

Name	Type	Size
Number	varChat	5
Name	varChat	10
Manager	varChat	20
Address	varChat	20
Phone number	varChat	50

According to the data in Table 3, we have systematically designed the attendance of employees of the enterprise. According to the E-R diagram, we also divide it into three categories for introduction in the attendance table. The first is the ID of the employee. The ID is generally set to a Bigint type with a length of 8 bits. The ID here is equivalent to positioning. When employees use the system to punch in, they will automatically locate, so the ID will change, which is also a way for companies to check employees’ attendance. The employee number is generally set to a 5-digit VarChat type, which is unique to each employee and cannot be changed by anyone. The most important thing about attendance is to clock in and clock out. They generally set a VarChat type with a length of 5 digits. After using the system to punch in, relevant information will be automatically generated to help the personnel department understand the attendance of employees. An important part of this structure is the remarks, this part is to solve the problem of other attendance situations, proportional field work, or the problem of not being able to clock in because of enterprise network problems. These situations can be remarked to address attendance issues.

**Table 3:** Attendance sheet related structure.

Name	Type	Size
ID	Bigint	8
Number	varChat	5
Name	varChat	10
Commuting status	varChat	5
Off duty status	Datetime	5
Date	Datetime	8
Remarks	varChat	10

According to the data in Table 4, we have systematically designed the salary situation of enterprise employees. According to the E-R diagram, time is an essential part of salary design. The month is generally set as a VarChat type with a length of 20 digits, which represents the salary of the employee in the month. If there are other related salaries that were not paid in full last month, they can be remarked in this section. Next is the number and name of the employee. This part is the same as the introduction above, and the employee number is unique. There are allowances and other types in the employee’s salary component, including leave, which involves bonuses and deductions other than the basic salary, and these contents are recorded in the corresponding parts of the system. These contents and the final paid salary are generally set to a 5-digit money type, and all information can be searched by raising the employee number in the salary part of the system.

**Table 4:** Structure related to payroll.

Name	Type	Size
Month	varChat	20
Number	varChat	5
Name	varChat	10
Basic salary	Money	5
Allowance	Money	5
Leave of absence	Money	5
Deductions	Money	5
Actual salary	Money	5

## Human resource management operation system

### Employee indicator data

In enterprise management, the management needs to analyze the basic situation of employees. In the process of analysis, it is mainly to investigate the relevant data indicators of employees. Enterprises include different departments, and employees may leave or newly join at different stages. We can intuitively show them according to the relevant functions of the system. The management can also analyze the management situation of the current department based on these data, so that the enterprise can develop in a more scientific direction.

**Figure 7:**
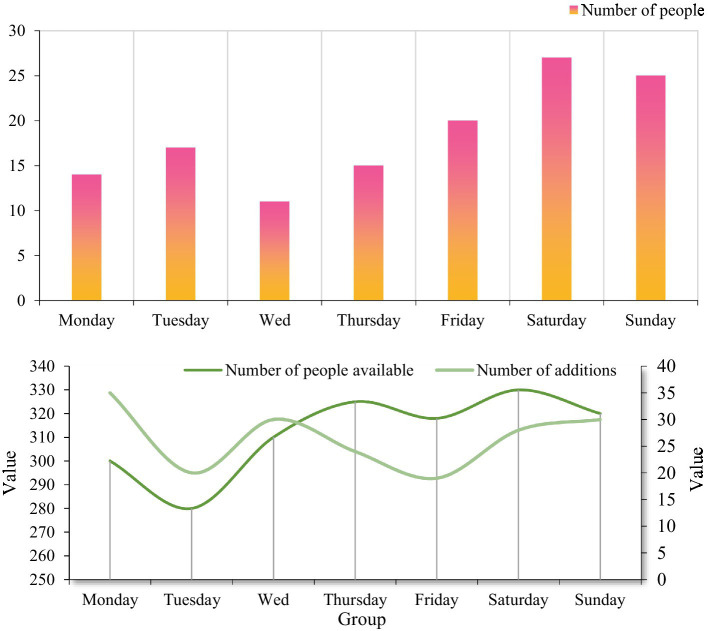
Description of employee resignation and job hunting.

We briefly introduced the relevant data of employees above. In Figure 7, we took Company A as an example to investigate the employee turnover of the formula. According to the data in the data classification, we divided it into resigned employees, incumbent employees, and new employees. First of all, from the perspective of employee turnover, we conducted a survey on the company’s turnover within a week. According to the survey data, the number of departures on Saturday is the largest, and the number of departures on Wednesday is the least. However, employee turnover occurs every day, and most on weekends. This may be related to the reality that most employees choose to leave on the weekend may have the basic work at hand completed. And it is free to schedule an interview next week.

Judging from the number of incumbent employees and new employees in the company, the number of employees in the first 2 days of the week is relatively small, but the overall number fluctuates little, which belongs to the normal flow of personnel. As the number of interviewers increases, the total number of people in the company will continue to increase. According to the survey data, the company has 330 employees on Saturday, the largest number in a week. Judging from the number of new employees, the number of new employees on Friday was the least, with only 19, and the most on Monday was 35. This situation is basically consistent with the actual situation, indicating that the records of the human resource management system are consistent with the facts, and we can use the data provided by the system to analyze the situation of the employees of the enterprise.

**Figure 8:**
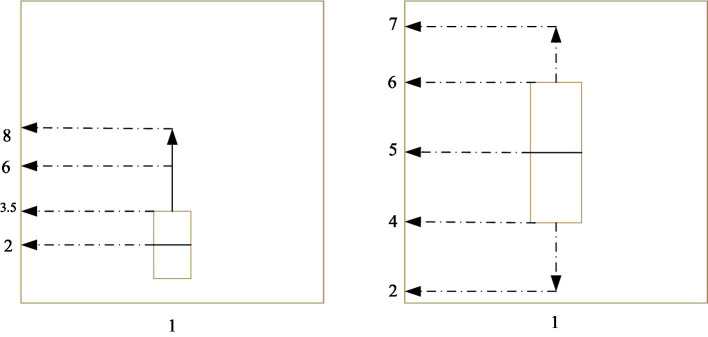
Employee working year analysis.

Employees are the most important resource of an enterprise. How to retain employees is essential to improve the development of the company. To this end, we analyzed the relationship between employee turnover and working years. According to the data in Figure 8, we have investigated the working years of the current and former employees of the company. First of all, we observe the on-the-job employees. The average working life of on-the-job employees is 3.5 years, and half of them have less than 2 years. From this data, it can be seen that the average number of years of on-the-job employees is short, and the work experience has yet to be accumulated.

Judging from the situation of resigned employees, the average age of resigned employees reached 5 years. Some of the employees are 4–5 years old, and some employees are 5–6 years old. According to this data, we know that most of the employees who leave are old employees. The “runaway” of these people is a large loss for the company. According to this situation, at present, the company’s labor turnover is relatively large, the working years of new employees are not enough, and the number of old employees is decreasing. Management needs to adjust company policies under objective conditions to retain employees. The situation of employees can be displayed very intuitively in the human resource management system. The management needs to analyze the relevant data in time, find countermeasures, and ensure that the formula develops in the direction of health science.

### Staff positions

As a relatively sound enterprise, the company will set up different positions to cooperate with the work. However, according to the situation of the century, the treatment of different positions is different, and the resignation of employees in different positions is also different. To investigate the resignation situation of employees in different positions, we used the human resource management system to summarize the in-service and resignation situations of employees in different positions. The details are as follows.

**Figure 9:**
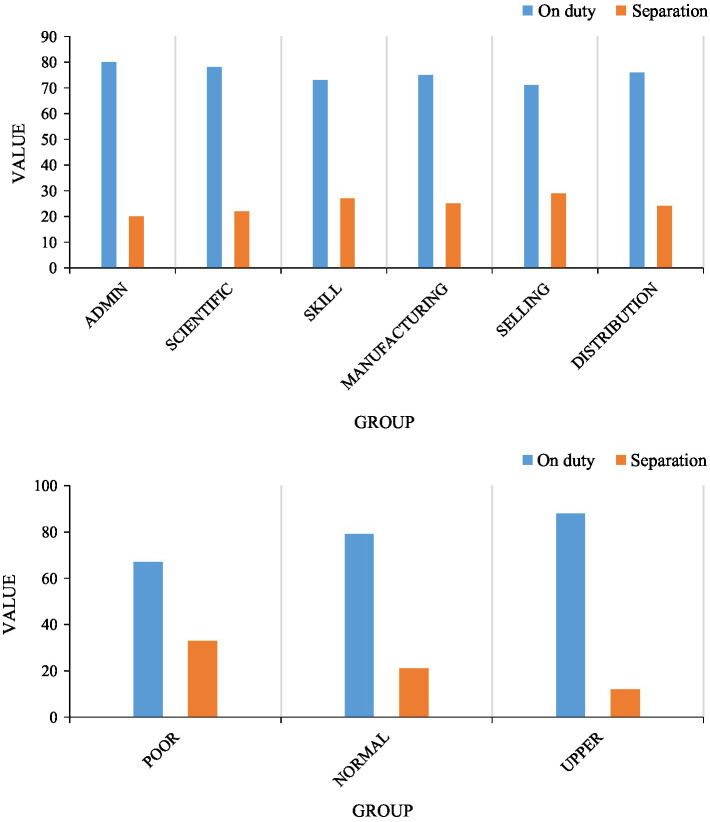
Analysis of employee positions.

Different positions have different attractiveness to employees. Some employees may like this job, and some people are looking forward to this salary. According to the data in Figure 9, we conducted a survey from the aspects of position and salary. We first analyze the situation from the position. According to the survey, the incumbency rate of management positions is self-high, reaching 80%, and the turnover rate is only 20%. It can be seen that the management positions of the company A are the most popular positions among all positions. It is followed by R&D positions and sales positions, and marketing positions have the lowest incumbency rate at 71%. From the overall situation, the incumbency rate of each position is relatively high, indicating that there is no shortage of employees in Company A. However, this data does not represent the quality of employees. It can be seen from the above that a large number of “runaways” of old employees is the most deadly.

From the perspective of salary, we conducted surveys from three dimensions: low salary, general salary, and high salary. According to the data survey, the incumbency rate of low salary is 67%, and the turnover rate is 33%; the incumbency rate of general salary is 79%, and the turnover rate is 21%; the incumbency rate of high salary is 88%, and the turnover rate is 12%. It can be seen that the turnover rate of low salary is the highest, and the turnover rate of high salary is the lowest, indicating that salary is an important factor affecting employees, and retaining employees can start from salary.

### Employee promotion situation

Employees need to seek progress in the enterprise, and if employees have been unable to get promoted, they may leave. Because the inability to be promoted may lead to the inability to raise wages, which will dampen the enthusiasm of employees and be detrimental to the normal development of the company. Therefore, enterprises need to formulate a scientific and reasonable job promotion system to stimulate the enthusiasm of employees. To investigate the promotion situation of employees, we conducted a survey on the operation projects and promotion situation of employees, and the details are as follows.

**Figure 10:**
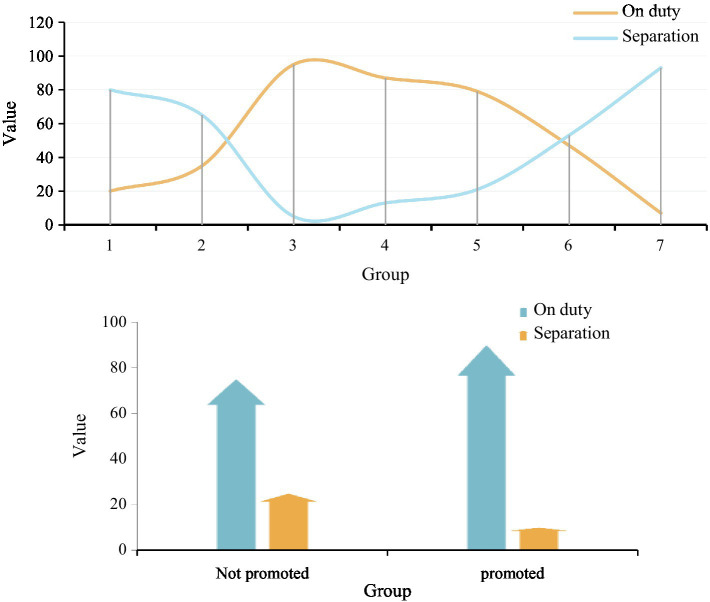
Analysis of employee promotions.

Projects and promotions of employees were investigated. According to the data in Figure 10, first of all, looking at the projects handled by employees, the incumbency rate of employees who only handle one project is 20%, and the turnover rate is 80%. 65% of employees who have handled the two projects have an on-the-job rate and a 35% turnover rate. The employees who have handled the three projects have an on-the-job rate of 95% and a turnover rate of 5%. From this situation, it can be seen that in the number of projects 1–3, the in-service rate of employees is increasing. In the number of projects 1–3, the on-the-job rate of employees is declining, and the turnover rate of employees is rising. Especially in the 7 projects handled, the on-the-job rate of employees is 7%, and the turnover rate is 93%. It can be seen from this that employee treatment is not the only factor that affects employees leaving the job. It may be due to external competition leading to brain drain. In response to this situation, companies need to take early countermeasures to reduce the occurrence of this situation.

From the perspective of employee promotion, according to the basic laws of the market, we conducted a survey on employee turnover in the experiment using one promotion every 3 years. According to the data, the incumbency rate of those who have not been promoted within 3 years is 75%, and the turnover rate is 25%; the incumbency rate of those who have been promoted within 3 years is 90%, and the turnover rate is 10%. According to the data, employees who have not been promoted within 3 years have a higher chance of leaving, which also shows that long-term lack of promotion is an important factor leading to employee turnover.

## Conclusion

With the continuous impact of the economy and the environment, the competition of enterprises is becoming increasingly fierce. If we want to maximize the benefits of enterprises by relying on high-tech means, we must closely follow the needs of technological change and establish a new management model. Therefore, this paper aims to study the organizational human resource management and operation system based on artificial intelligence. It expects to use artificial intelligence technology to design human resource management systems. It improves the quality of employees so that the enterprise develops toward a more scientific and reasonable method. Experimental investigations have found that compensation, performance, and promotion are effective ways to manage employees. Especially in terms of salary management, it needs to be adjusted in time according to the actual situation, and at the same time, it is necessary to formulate employee promotion channels to meet the development needs of employees. Enterprises can use the human resource management system to understand the situation of employees in a timely manner and adjust the management policies of the enterprise in time. By continuously improving the strength of the workforce, they work together to forge ahead with the company’s development goals. Although this paper has some conclusions in the process of exploration, there are still shortcomings: the operating efficiency of the system needs to be improved, and the system is not efficient enough when dealing with a large amount of data.

## Data availability statement

The original contributions presented in the study are included in the article/supplementary material; further inquiries can be directed to the corresponding author.

## Author contributions

YY has participated in conception and design, analysis and interpretation of the data, drafting the article, and revising it critically for important intellectual content.

## Funding

Supported by the high level talent scientific research startup foundation of North China University of Water Resources and Electric Power (No. 201801004).

## Conflict of interest

The author declares that the research was conducted in the absence of any commercial or financial relationships that could be construed as a potential conflict of interest.

## Publisher’s note

All claims expressed in this article are solely those of the authors and do not necessarily represent those of their affiliated organizations, or those of the publisher, the editors and the reviewers. Any product that may be evaluated in this article, or claim that may be made by its manufacturer, is not guaranteed or endorsed by the publisher.
